# Loss of charge mutations in solvent exposed Lys residues of superoxide dismutase 1 do not induce inclusion formation in cultured cell models

**DOI:** 10.1371/journal.pone.0206751

**Published:** 2018-11-06

**Authors:** Keith Crosby, Anthony M. Crown, Brittany L. Roberts, Hilda Brown, Jacob I. Ayers, David R. Borchelt

**Affiliations:** 1 Department of Neuroscience, Center for Translational Research in Neurodegenerative Disease, University of Florida, Gainesville, Florida, United States of America; 2 College of Arts and Sciences, University of Florida, Gainesville, Florida, United States of America; 3 SantaFe HealthCare Alzheimer’s Disease Research Center, McKnight Brain Institute, University of Florida, Gainesville, Florida, United States of America; Children’s Hospital of Pittsburgh, University of Pittsburgh Medical Center, UNITED STATES

## Abstract

Mutations in superoxide dismutase 1 (SOD1) associated with familial amyotrophic lateral sclerosis (fALS) induce the protein to misfold and aggregate. Missense mutations at more than 80 different amino acid positions have been associated with disease. How these mutations heighten the propensity of SOD1 to misfold and aggregate is unclear. With so many mutations, it is possible that more than one mechanism of aggregation may be involved. Of many possible mechanisms to explain heightened aggregation, one that has been suggested is that mutations that eliminate charged amino acids could diminish repulsive forces that would inhibit aberrant protein:protein interactions. Mutations at twenty-one charged residues in SOD1 have been associated with fALS, but of the 11 Lys residues in the protein, only 1 has been identified as mutated in ALS patients. Here, we examined whether loss of positively charged surface Lys residues in SOD1 would induce misfolding and formation of intracellular inclusions. We mutated four different Lys residues (K30, K36, K75, K91) in SOD1 that are not particularly well conserved, and expressed these variants as fusion proteins with yellow fluorescent protein (YFP) to assess inclusion formation. We also assessed whether these mutations induced binding to a conformation-restricted SOD1 antibody, designated C4F6, which recognizes non-natively folded protein. Although we observed some mutations to cause enhanced C4F6 binding, we did not observe that mutations that reduce charge at these positions caused the protein to form intracellular inclusions. Our findings may have implications for the low frequency of mutations at Lys residues SOD1 in ALS patients.

## Introduction

Amyotrophic Lateral Sclerosis (ALS) is a fatal neurodegenerative disease primarily characterized by loss of upper and lower motor neurons. Although most forms of ALS are of unknown etiology (sporadic ALS), a subset of cases demonstrate dominant patterns of inheritance in specific proteins (familial ALS or fALS). Of these inherited genetic mutations, approximately 20% are found in Cu-Zn superoxide dismutase (SOD1) [1], the ubiquitous antioxidant protein responsible for metabolizing oxygen radicals in the cytoplasm [2,3]. SOD1 is a homodimer composed of 153-amino acid subunits in which each subunit contains eight β-strands, a catalytic copper ion, a structurally important zinc ion, an electrostatic loop element that forms a portion of the active site funnel, and an intramolecular disulfide bond between cysteine 57 and cysteine 146 [4–6]. Over 160 mutations in SOD1 have been associated with ALS {http://alsod.iop.kcl.ac.uk/default.aspx}. Disease onset for SOD1-fALS patients is 45–47 years [7], whereas the average age of onset in sALS cases tends to be later (55–60 years of age) [8].

The vast majority of SOD1 mutations associated with ALS are missense point mutations. The effects of fALS mutations on the normal enzyme activity and protein turnover vary greatly [9–13]. While some mutants are rapidly degraded or inactive, others retain high levels of activity and relatively long half-lives [9–18]. SOD1 with mutations associated with fALS is generally viewed as being more prone to misfold and aggregate [7,14,19–22]. SOD1 immuno-reactive inclusions in surviving spinal motor neurons is a common, but not uniformly found, pathologic feature of SOD1-linked fALS [23–38]. Notably, the SOD1 inclusions found in patients appear to lack the features of amyloid (Thioflavin and Congo Red negative) [23,39]. Misfolded SOD1 has also been described as a pathologic feature of sporadic ALS using antibodies that are preferentially reactive to non-natively folded SOD1 [40–42]. However, other studies have disputed these findings [43–45]. Thus, although the role of wild-type SOD1 in sporadic ALS requires further study, there is substantial evidence of misfolded and aggregated SOD1 in fALS patients with SOD1 mutations (reviewed in [46]).

In cell culture and mouse models of WT and mutant SOD1 expression, there is a clear distinction between the aggregation propensities of WT and mutant protein. Although mice expressing high levels of human WT SOD1 develop clinical signs of ALS, including paresis, the age at which WT mice reach end-stage is approximately 2 times longer than mice that express comparable levels of G93A fALS SOD1 (367±56 days vs 155±9 days) [47]. At end-stage, the levels of mis-folded, aggregated SOD1 detected by filter-trap assay [21] were two-fold higher in paralyzed G93A mice than in paralyzed WT over-expressing mice [47]. Mice expressing WT-SOD1 fused to yellow fluorescent protein (SOD1:YFP) age normally and show little or no evidence of WT-SOD1:YFP aggregation; whereas equivalently expressed fALS mutant G85R-SOD1:YFP produces clinical signs of ALS with evidence of mutant protein oligomerization, aggregation, and inclusion formation [48]. In cell culture studies of SOD1 aggregation, we have consistently observed that WT SOD1 is >10-fold less prone to form detergent-insoluble aggregates than fALS mutant SOD1 (>40 mutants tested) [7,49,50]. These studies use a paradigm of transient over-expression in which aggregation of mutant SOD1 occurs over a period of 24 to 48 hours. The aggregates generated in these cell models are similar to what is generated in transgenic mouse models expressing mutant SOD in that in both cases the protein that acquires detergent-insolubility lacks a normal intramolecular disulfide bond [51].

Similar transient expression paradigms have been used to visualize the aggregation of mutant SOD1 in cultured cells, using a strategy in which SOD1 is fused to a fluorescent reporter protein [52–58]. In cells expressing mutant SOD1 fused to the fluorophore, fluorescent inclusions were observed; whereas WT SOD1 fused to fluorophore was diffusely distributed throughout the cytosol. We have demonstrated that WT and mutant SOD1 fusions proteins with YFP show differing propensities for inclusion formation, differences in detergent solubility, and differences in diffusibility in disrupted cells [59]. SOD1:YFP fusions with fALS mutations A4V, G37R, G85R, D101N, C111Y, and S134N all readily form fluorescent inclusions when over-expressed [59,60]. Untagged versions of SOD1 with these same mutations form detergent insoluble, sedimentable, aggregates when transiently over-expressed [7,50]. Moreover, in studies that have experimentally combined familial and experimental mutations in SOD1 to examine the role of disulfide cross-linking in aggregation, we have observed complete agreement between the aggregation propensities of untagged and YFP tagged SOD1 variants [60]. Collectively, this body of work demonstrates that fALS mutations in SOD1 share a common feature of promoting aggregation of the protein, and that visualizing inclusion formation by expressing YFP fusion proteins is a useful approach.

**Fig 1:**
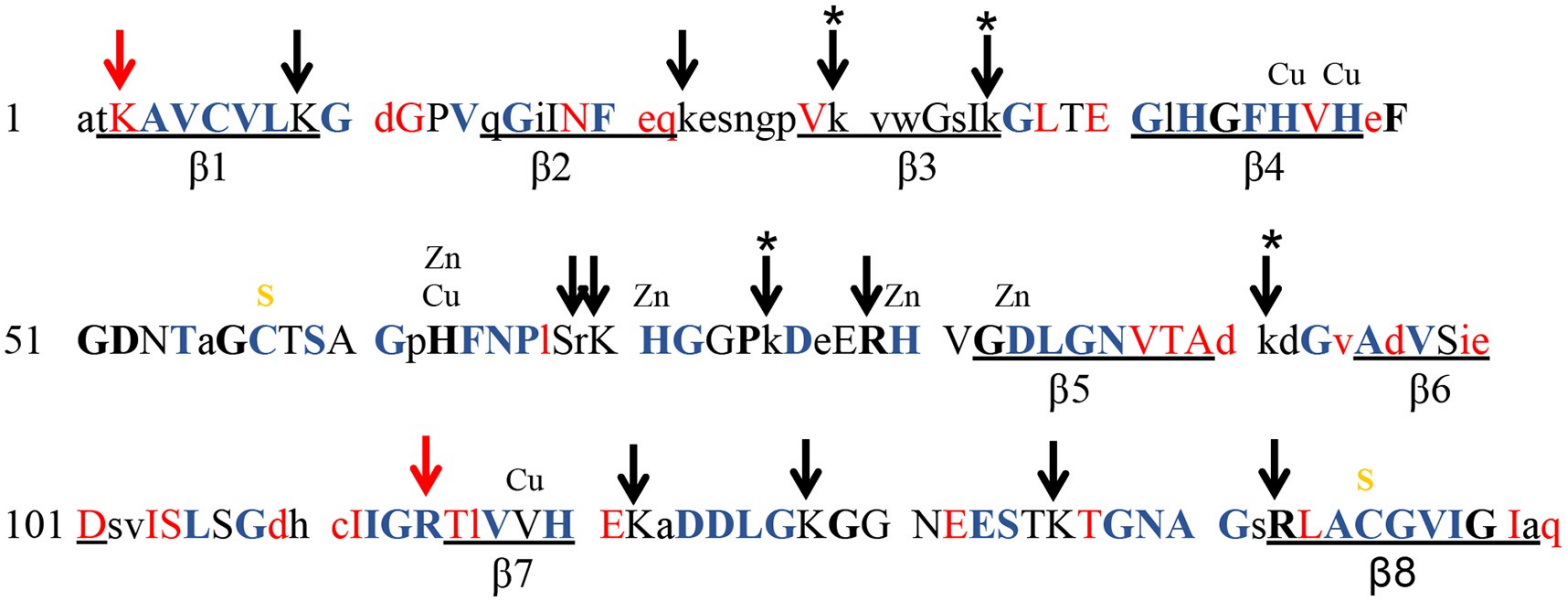
Location of mutations in SOD1 that have been identified in patients with ALS {http://alsod.iop.kcl.ac.uk}. Purple, bold, font indicates a position where a point mutation occurs at a residue that is highly conserved across multiple species. Red letter font indicates a position where a point mutation has been identified in ALS patients (Capitalized—conserved in mammals; lower case—not conserved). The positions of Cu and Zn binding are marked as are two Cys residues involved in an intramolecular disulfide bond (yellow S). Arrows mark the positions of Arg and Lys residues (Red arrow notes position with known ALS mutations). One of the four Arg residues and one of the eleven Lys residues are positions with known point mutations at time of writing. This figure is an adaptation of a figure originally published in [65].

The objective of the current study was to investigate whether loss of positively charged residues in SOD1 induce the aggregation of SOD1. It has been proposed that one mechanism by which mutations in proteins could enhance propensity to aggregate is by reducing the repulsive forces that could occur when charged residues try to align and stack in aberrant aggregates, or when two monomers of misfolded protein interact in prelude to the formation of stronger aberrant interactions [61–63]. In examining the frequency of mutations in positively charged residues of SOD1 (Fig 1, arrows), we noted that only one of 11 Lys (K3 –red arrow) and one of four Arg (R115 –red arrow) residues are known mutation sites in ALS {http://alsod.iop.kcl.ac.uk/default.aspx} (Fig 1, red font indicates positions of known ALS mutations). Five of seven His residues are sites of mutation; however, these amino acids play critical roles in the binding of metal cofactors that contribute to the stability of native SOD1 structure [64]. By contrast, nine of 11 Asp and five of nine Glu residues (negatively charged) have been identified as sites of mutation in ALS {http://alsod.iop.kcl.ac.uk/default.aspx} (Fig 1). Several of the mutations associated with ALS that occur at His, Asp, and Glu residues have been examined in different types of aggregation paradigms, and all of the mutants show a higher propensity to aggregate [7]. Although the aggregation data on mutations that occur in negatively charged residues are consistent with the idea that loss of repulsive forces could contribute to mutant SOD1 aggregation, the paucity of mutations in positively charged residues is curious.

To determine whether simple loss of positive charge at Lys residues may promote SOD1 misfolding, and hence promote inclusion formation, we introduced amino acid substitutions at four Lys residues that are not particularly well conserved (Fig 1, positions marked by asterisk). The rationale being that more conserved Lys residues might be critical to a structural feature of the protein whereas less conserved residues would provide a better test of whether simply altering the overall charge of the protein could induce inclusions to form. The charged R-group for all four of these Lys residues is predicted to be solvent exposed based on the X-ray crystallographic structure pdb 1HL5 (S1 Fig) [66]. Our objective was to determine whether simply altering charge at these non-conserved Lys residues would cause the protein to produce intracellular inclusions by over-expressing SOD1:YFP fusion proteins. In prior studies, we have used both HEK293 and Chinese Hamster Ovary (CHO) cells to visualize the formation of inclusions by mutant SOD1 fused to YFP, finding similar results between the two cell lines [59,60]. In these prior efforts, we have validated that when inclusions form in these cells, the structures do not diffuse out of cells that have been treated with membrane permeablizing agents (e.g. saponin), and that cultures with inclusions have higher levels of detergent insoluble mutant SOD1:YFP fusion protein [59]. Here we have used the CHO model largely because these cells tend to be flat with a large cytosolic compartment that allows for a clearer assessment of whether inclusions are present. An important aspect of this approach is that the SOD1:YFP proteins are highly over-expressed. In this setting any modulation of inclusion formation by chaperone function or variabilities in protein stability are minimized, revealing inherent propensities of the protein to form inclusion structures [7,67]. In these over-expression model systems, both WT and mutant SOD1 are largely deficient in Cu^2+^ ions and are less able to form the normal intramolecular disulfide bond associated with full maturation [67]. Thus, the model is essentially assessing the inherent propensity of immature mutant SOD1 to achieve native conformation. Our experimental data with this model indicates that loss-of-charge mutations in solvent exposed Lys residues are not sufficient to induce SOD1 to spontaneously form intracellular inclusions.

## Materials and methods

### Generation of mutant SOD plasmids

Human SOD1 cDNA’s with mutations described in this study were generated using oligonucleotide primers encoding the desired mutation with Quick-Change mutagenesis kits. The single mutants were made using pEF.BOS vectors [68] that encode WT human SOD1 (WT-hSOD1) as the template [49]. The protocol used to make mutations used a modified PCR strategy with primers encoding specific mutations and pEF-BOS-WT SOD1 or pEF-BOS-WT SOD1:YFP plasmids as the template. The PCR reaction used Platinum *Pfx* polymerase (Invitrogen/ThermoFisher, Waltham, MA) and 2X Pfx buffer concentration to accommodate the large plasmids that were amplified. The PCR reaction products were digested with Dpn1 to remove template and then transformed into NEB-10β competent cells (New England Biolabs, Ipswich, MA) following standard protocols. Large scale preparations of plasmid DNA for transfection were prepared by CsCl gradient purification. The SOD1 and SOD1:YFP coding sequence of all plasmids was verified by DNA sequence analysis.

### Transient transfections

Plasmid DNA encoding the mutant SOD1:YFP cDNAs were transiently transfected into Chinese hamster ovary (CHO) cells. The day before the transfection, the CHO cells were split into 60-mm poly-D-lysine-coated dishes (1 plate for each DNA construct). Upon reaching 95% confluency, cells were transfected with Lipofectamine 2000 (Invitrogen/ThermoFisher). The cells were then incubated at 37°C in a CO_2_ incubator for 24 hours at which time images of random fields of view at 20x and 40 x magnification were captured using an AMG EVOS_fl_ digital inverted microscope for fluorescence. The cells were returned to the incubator for 24 hours before images were captured again. The transient transfections were repeated at least 3 times for each construct. The images from multiple transfections were analyzed and the following objects were counted in a blinded fashion; cells showing YFP fluorescence and fluorescent cells that exhibit multiple inclusion puncta.

### Immunocytochemistry

The day before transfection, CHO cells were plated onto poly-D-lysine coated glass coverslips. 24 or 48 hours following transient transfection with pEF-BOS-SOD1 constructs, cells were fixed with 4% paraformaldehyde for 10 minutes at room temperature. Following washing with 1X PBS, cells were perforated with ice-cold methanol. Non-specific binding was blocked with 5% normal goat serum for 1 hour at room temperature and then incubated with the mouse monoclonal antibody C4F6 [69], diluted 1:1000 in 3% normal goat serum, at 4 degrees Celsius overnight. Following PBS washes, cells were incubated with goat anti-rabbit AlexaFlour-488 and goat anti-mouse AlexaFlour-568 (Invitrogen) at 1:2000 in 3% normal goat serum at room temperature for 1 hour. In some cases, we included a 1:2000 dilution of DAPI in the incubation with secondary antibody. Cells were washed with PBS and mounted to glass slides (Fisher) with Aqua-Poly/Mount coverslipping medium (Polysciences, Inc, Warrington, PA). Fluorescent microscopy images were captured on an Olympus BX60 epifluorescence microscope. Most images were captured at 20x.

### Immunoblotting

At 48 hours post-transfection, CHO cells were washed from the plate in 1X PBS, and then centrifuged at 3000xg rpm for 5 minutes before resuspension in 1X PBS with protease inhibitor cocktail (Sigma, St. Louis, MO, USA). Cells were disrupted with a probe sonicator for 10 seconds, and protein concentrations determined with BCA assay (Pierce/ThermoFisher, Waltham, MA). 5μg of total protein was loaded and separated through an 18% TG-SDS PAGE gel (Invitrogen/ThermoFisher) and transferred to nitrocellulose membrane. The membranes were incubated in Odyssey blocking solution (Li-Cor, Lincoln, Nebraska) as directed by the manufacturer and then primary antibodies (SOD1 whole protein rabbit polyclonal antibody [70] and C4F6 monoclonal antibody) were incubated at 1:2000 overnight in Odyssey blocking solution. The membranes were washed in 1X TBS-T, and probed with goat anti-rabbit IRDye-680RD and goat anti-mouse IRDye-800CW near-infrared-labeled secondary antibodies (Li-Cor). Images were captured using an Odyssey imaging system (Li-Cor) and densitometry analysis was performed using ImageJ (NIH, Bethesda, MD). The values for the intensity of SOD1 bands were normalized to G93A SOD1 protein and the data were graphed using Excel.

## Results

The 4 four non-conserved positions encoding Lys we chose to mutate were altered to introduce residues that are found at the cognate position of SOD1 in other species. The four mutants tested here were K30V, K36T, K75A, and K91G. The amino acids in mouse SOD1 at the positions corresponding residues 30, 36, and 75 are V, T, and A, respectively. The amino acid encoded at position 91 is more highly conserved in mammals and for this residue we chose to introduce Gly to diminish the likelihood of disrupting flexibility around this position, which is located in a critical loop structure between beta strands 5 and 6. Notably, a Gly residue is found the cognate position of SOD1 in *Drosophilia melanogaster*, *Candida albicans*, and *Cryptococcus neoformans* (see Supporting Information in [65]). The mutations introduced at these positions are similar to the types of substitutions reported at other positions in patients with ALS (Valine, threonine, alanine, and glycine substitutions have been reported at multiple positions: e.g. A4V, L8V, A4T, I113T, D11A, G93A, C6G, and D101G to name a few {http://alsod.iop.kcl.ac.uk/}). Thus, in each case studied here, the substitution introduced was a type of change that has been reported in ALS patients while also introducing a residue that would be found at the cognate position of SOD1 in another species. The goal was to eliminate the charged lysine residues without introducing an amino acid substitution that might be highly destabilizing independent of the potential influence of loss-of-charge.

**Fig 2:**
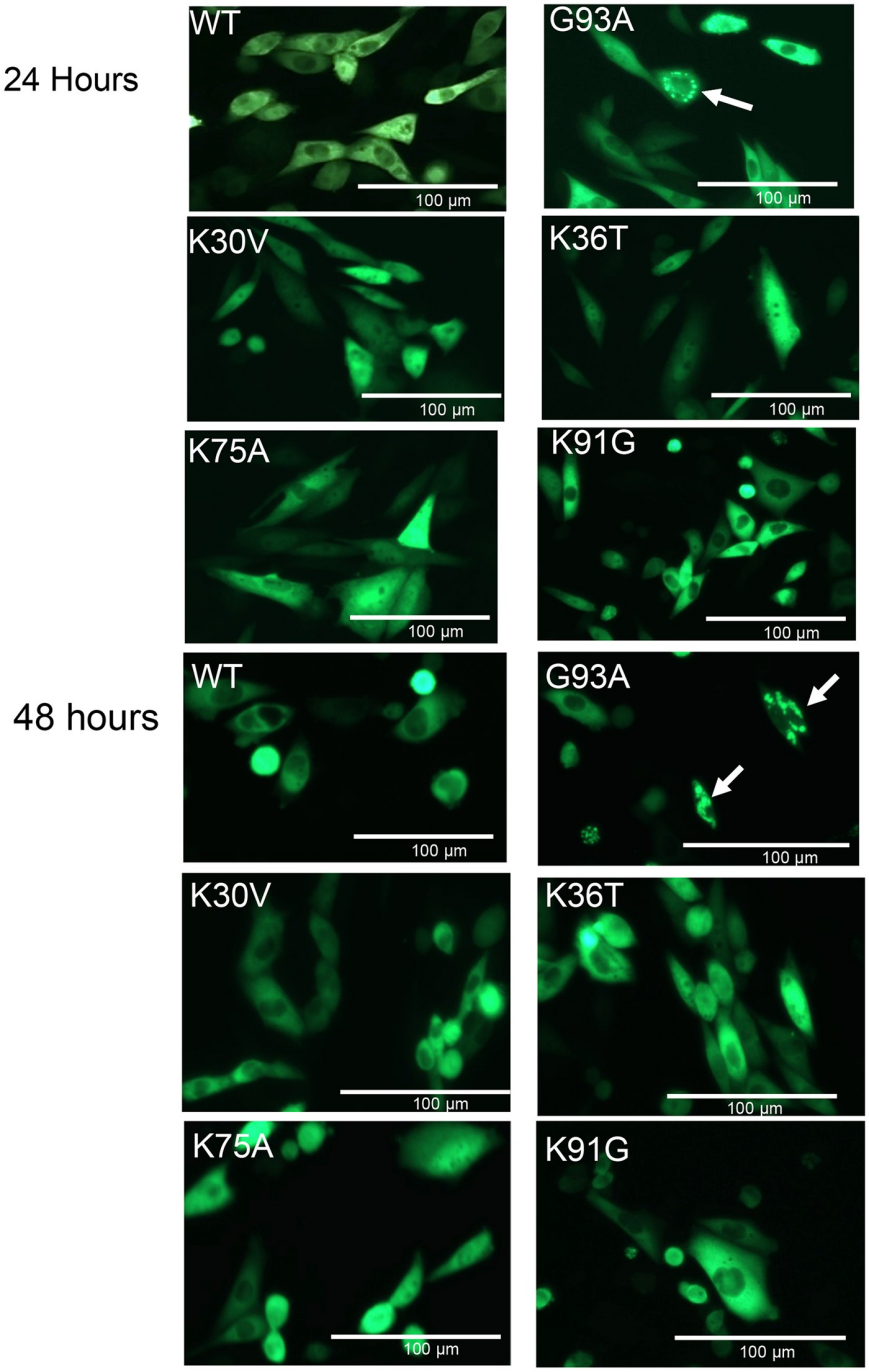
Cell assay of inclusion formation by SOD1 variants encoding mutations at lysine residues. CHO cells were transfected with plasmids for each SOD1:YFP variant and representative pictures of direct YFP fluorescence were taken at 24 and 48 hours post-transfection, using a conventional epifluoresence microscope (20x magnification). G93A-SOD1:YFP provides a positive control for inclusion formation and WT provides a negative control for inclusion formation. The percentage of cells expressing these mutants (K30V, K36T, K75A, or K91G) that developed inclusions was far lower than the positive control G93A variant (Table 1).

As described in the Introduction, we assessed whether SOD1 with these mutations, fused to YFP, would cause the protein to form intracellular inclusions [59]. In this paradigm, fluorescence intensity can be used as a gauge for relative expression. As described in Methods, three transient transfections were performed for each construct and representative images were captured at 24 and 48 hours. For quantitative analysis of aggregate formation, cells expressing the fusions proteins were identified by fluorescence and scored for the presence of inclusion-like structures. The criteria for scoring was to identify cells in which the overall flat morphology of the cell is preserved so that the observer can discern whether the YFP fluorescence is diffusely distributed, or if the fluorescence is coalesced into more intensely fluorescent puncta indicative of insoluble inclusions [59]. Cells must have multiple puncta to score as positive (Fig 2; arrows in G93A panels identify cells examiners used as positive control examples). In prior studies of cells expressing mutant SOD1:YFP fusion proteins, we observed that many different ALS variants of SOD1 produce similar profiles of inclusion pathology (either encircling the nucleus or distributed through the cytosol). Random fields of view were captured as images that were coded for a blinded observer to score, using a reference image of cells expressing G93A-SOD1:YFP for comparison (Table 1). Similar to our previous work with this approach [59,60], a subset of cells adopted a condensed, rounded, morphology and these cells were not scored because the center of these cells is out of the focal plane and it is difficult for the blinded observer to easily discern whether they contained inclusions. Prior studies have shown that these condensed, rounded, cells do not display markers associated with cell death [TUNEL labeling or dimeric Ethidium bromide uptake [59]], and the basis for their appearance is unknown. The percentage of cells that became rounded appeared to be approximately the same with transfection of vectors for WT-SOD1:YFP, and thus, the cell rounding was not specific to mutant SOD1:YFP expression (also see [59,60]).

**Table 1:** Frequency of inclusions and C4F6 reactivity.

SOD1 Variant	# cells counted (24 hr)	# cells with inclusions (24 hr)	% cells with inclusions (24 hr)	# cells counted (48 hr)	# cells with inclusions (48 hr)	% cells with inclusions (48 hr)
WT	492	3	≤ 1%	742	6	1%
G93A	380	196	52%	503	243	48%
K30V	431	15	3.4%	618	25	4%
K36T	550	13	2.4%	736	29	4%
K75A	377	9	2.4%	530	26	5%
K91G	207	1	≤ 1%	123	4	3%
K91N	208	14	7%	113	2	2%
K91R	176	2	1%	167	0	0%
SOD1 Variant	# cells counted (24 hr)	# cells with C4F6 reactivity (24 hr)	% cells with C4F6 reactivity (24 hr)	# cells counted (48 hr)	# cells with C4F6 reactivity (48 hr)	% cells with C4F6 reactivity (48 hr)
WT	114	13	11%	415	9	2%
G93A	419	370	88%	340	307	90%
K30V	n.d.	n.d.	n.d.	145	31	21%
K36T	n.d.	n.d.	n.d.	164	14	9%
K75A	n.d.	n.d.	n.d.	96	15	16%
K91G	207	25	12%	171	10	5%
K91N	251	141	56%	350	99	28%
K91R	187	31	16%	127	23	18%

n.d. = not done

**Fig 3:**
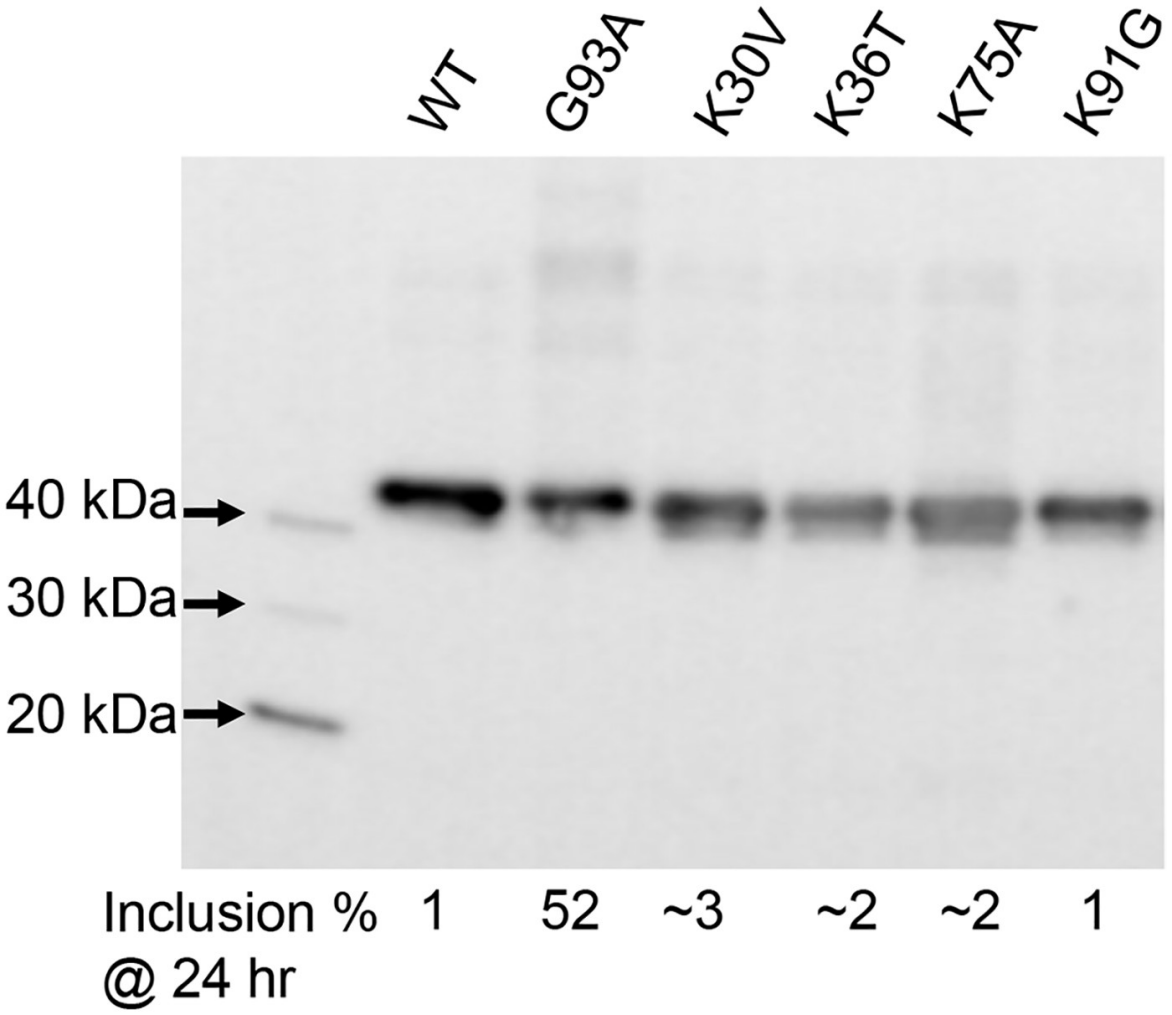
Immunoblot analysis of cells expressing WT and mutant SOD1 fused to YFP. CHO cells were transfected with plasmids for each SOD1:YFP variant and harvested at 24 hours for immunoblot analysis with antibody to SOD1 (see Methods). Each lane contains 5 μg of total proteins (measured by BCA assay—see Methods). Despite similar levels of expression, the percentage of cells expressing the K30V, K36T, K75A, or K91G mutants that developed inclusions was far lower than the positive control G93A variant (see Table 1). The image shown is representative of 3 independent immunoblotting experiments. At the level of expression and exposure shown here, endogenous CHO SOD1 is not visible.

**Fig 4:**
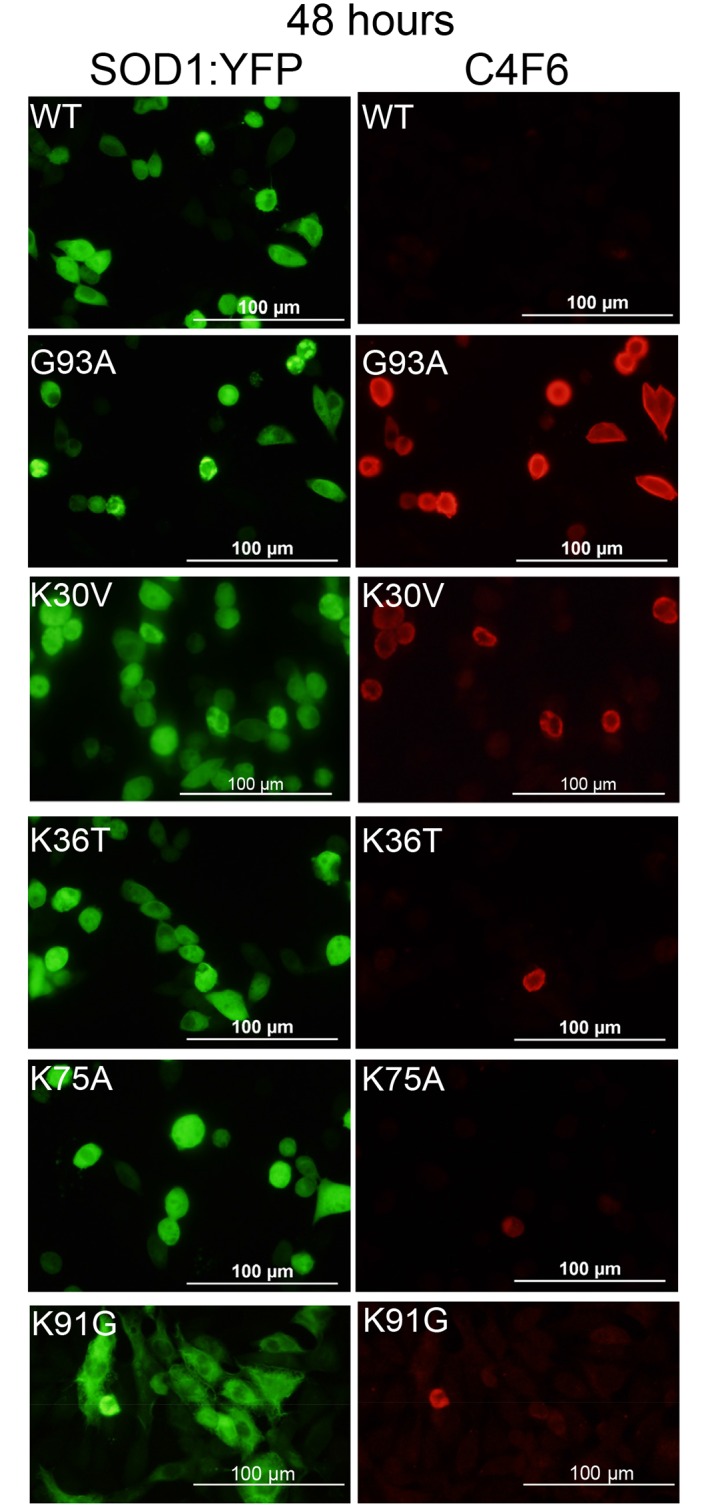
Analysis of C4F6 binding by SOD1 variants encoding mutations at Lys residues. CHO cells were transfected with plasmids for each SOD1:YFP variant and after 48 hours the cells were fixed and immunostained as described in Methods. One image of direct YFP fluorescence was captured before a second image of C4F6 immunoreactivity (red) was captured, using a conventional epifluoresence microscope (20x magnification). Cells transfected with WT-SOD1:YFP serve as a negative control and cells transfected with G93A-SOD1:YFP serve as a positive control. Each transfection was repeated at least 3 times and at least 3 fields of view were counted for cells expressing the YFP fusion protein that were also labeled with C4F6 (Table 1).

Focusing on cells with a flatter morphology, mutations at four different Lys positions (K30V, K36T, K75A, K91G) produced very few cells with inclusions at either time point (Fig 2; Table 1). Immunoblotting of cell lysates confirmed that the expression levels of these fusion proteins was similar to that of the positive control G93A-SOD1:YFP variant (Fig 3). In addition to assessing the effect of mutations on SOD1 inclusion formation, we examined whether the mutations may produce more subtle effects on folding, using the binding of C4F6 antibody as an assay [44,59,69,71]. The epitope for C4F6 has been partially defined as including amino acids D90, D92, G93,and D96 of the protein segment 90DKDGVAD96 [44,71]. When C4F6 is used in immunocytochemistry of fixed cells transiently over-expressing mutant SOD1, cells expressing many different fALS mutants show much stronger reactivity than cells expressing WT protein [44,59]. Importantly mutations at residues located relatively far from the C4F6 binding site, such as the A4V mutation, enable the binding of C4F6 [59]. Thus, C4F6 can be used as a sensitive detector of changes in SOD1 conformation when used in immunocytochemistry. As compared to cells expressing G93A-SOD1, cells expressing K30V, K36T, K75A, or K91G variants showed a lower frequency of C4F6 immunoreactivity (Fig 4; Table 1). The K91G mutant produced the lowest frequency of C4F6 binding, but because this residue is within the epitope recognized by C4F6 (second amino acid) we were uncertain as to whether the mutation lowered antibody affinity.

**Fig 5:**
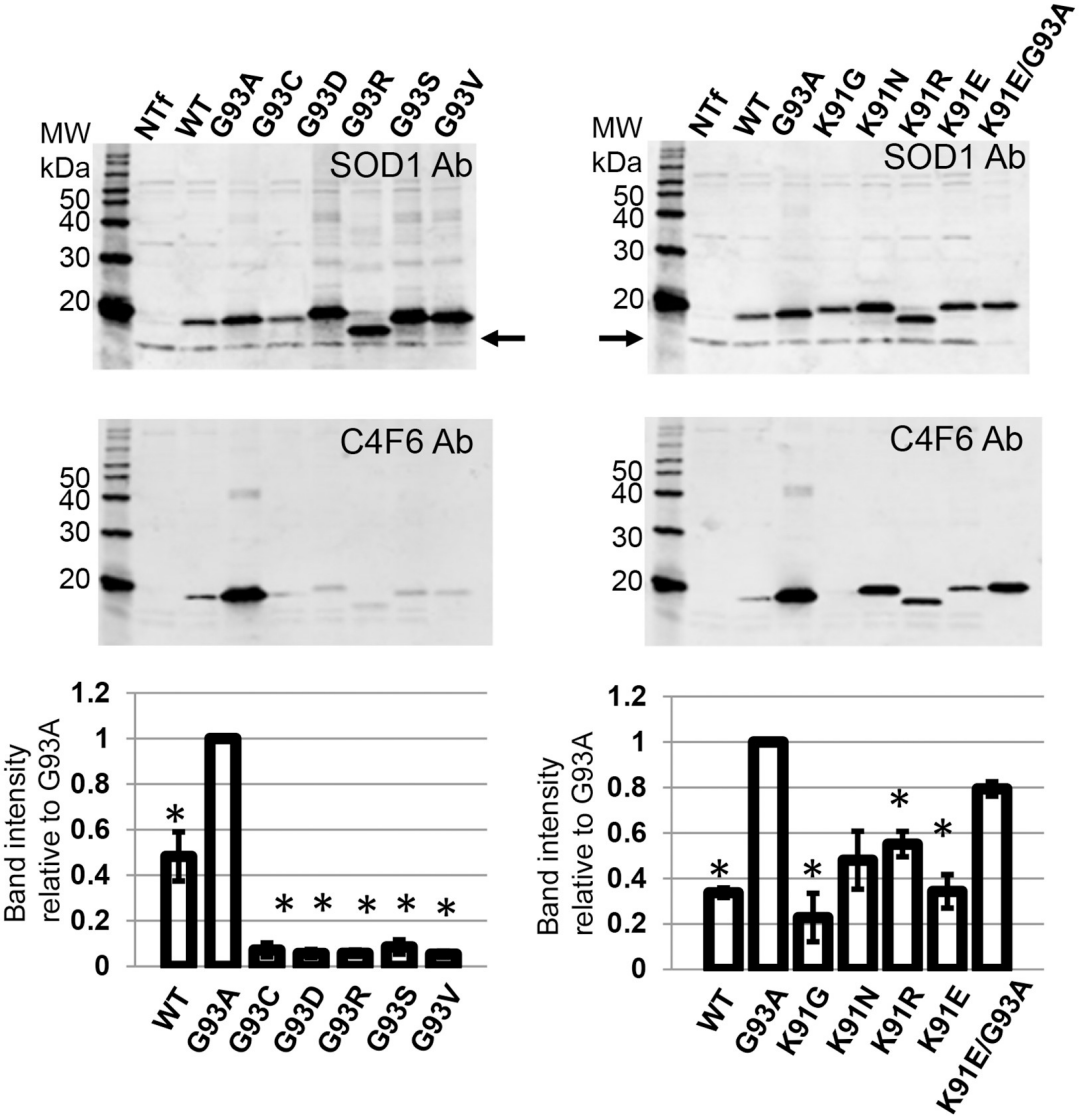
Immunoblot of cell lysates from CHO cells transfected with SOD1 variants encoding fALS mutations at Gly 93. The cells expressing these SOD1 variants (untagged) were harvested at 48 hours post-transfection, lysed, and analyzed by immunoblotting with the C4F6 and SOD1 antibodies as described in Methods. Each lane of the gel was loaded with 5μg of total protein. For each blot we quantified the band intensity (arbitrary units) and normalized the data to the value for G93A. The normalized values for 3 each individual blots were averaged and graphed. The binding of C4F6 to denatured SOD1 was greatly reduced if G93 was mutated to other residues that cause fALS, including G93C, G93D, G93R, G93S, and G93V (* p<0.05 G93A versus all other variants, T-Test, 2 tailed, unequal variance). When position 93 is Gly, the affinity of C4F6 is inherently weak but still detectable. The G93C, G93D, G93R, G93S, and G93V variants were all less reactive than WT SOD1 to C4F6 (p<0.05, T-Test, 2 tailed, equal variance). The arrows in the upper panels mark the positon of endogenous CHO SOD1. SOD1 with mutations of K91 to G, R, or E were also less reactive to C4F6 than G93A SOD1 (* p<0.05 G93A versus all other variants, T-Test, 2 tailed, unequal variance). Reactivity to the K91E/G93A double mutant was not significantly different from G93A SOD1. Reactivity of C4F6 to K91N SOD1 trended to significance, but higher variability in the data for this mutant caused the *p* value to be above the threshold for significance.

**Fig 6:**
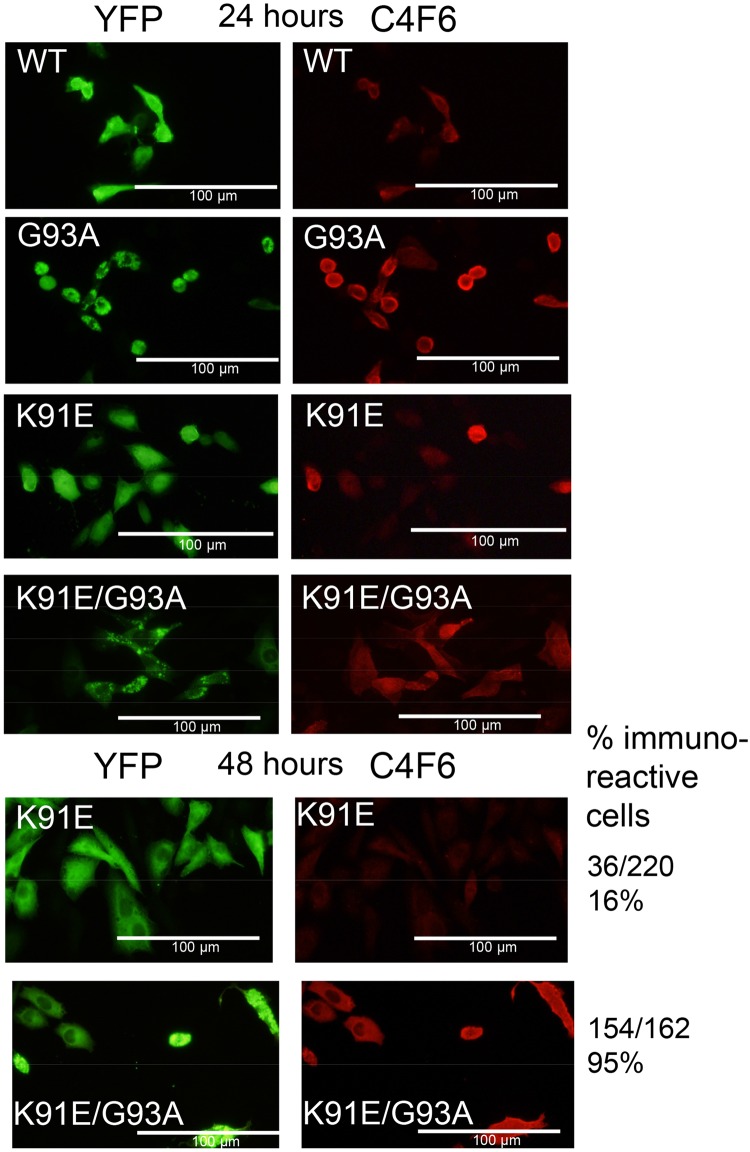
Analysis of C4F6 binding by SOD1 variants encoding an experimental K91E mutation in G93A hSOD1. CHO cells were transfected with plasmids for each SOD1:YFP variant and after 24 or 48 hours the cells were fixed and immunostained as described in Methods. One image of direct YFP fluorescence was captured before a second image of C4F6 immunoreactivity (red) was captured, using a conventional epifluoresence microscope (20x magnification). Cells transfected with WT-SOD1:YFP serve as a negative control and cells transfected with G93A-SOD1:YFP serve as a positive control (see Fig 4 for an example of 48 hours post-transfection). Each transfection was repeated at least 3 times. For the 48 hour time point, we captured images from at least 3 fields of view and counted all cells expressing the YFP fusion protein and cells that were also labeled with C4F6. Mutation of Lys 91 to Glu in G93A-SOD1:YFP did not markedly diminish the frequency of cells binding the C4F6 antibody at 48 hours post-transfection.

To define the C4F6 epitope further, we examined its reactivity to a panel of SOD variants by immunoblotting. The C4F6 antibody was raised against recombinant human G93A SOD1 and will bind to denatured protein in immunoblots [69]. The nature of the conformational specificity of C4F6 is not completely understood, but it appears that the antibody binds an epitope inclusive of residues D90 to D96 that is normally inaccessible in WT SOD1 [44]. In immunoblot analysis of a panel of ALS variants at the G93A position (G93A, G93C, G93D, G93R, G93V), it was clear that the antibody exhibited the greatest binding to denatured G93A SOD1, with moderate levels of reactivity to denatured WT (G93) protein (Fig 5). The ability of the antibody to recognize denatured WT SOD1 is consistent with the idea that this epitope is inaccessible in natively folded protein (detected in immunohistochemistry in fixed cells), and that mutations distal to the G93 position enable C4F6 binding by altering the protein conformation around the D90 to D96 epitope [44]. To determine whether K91 was critical in C4F6 binding, we generated a panel of mutations at Lys 91 (K91G, K91N, K91R, and K91E). The K91G variant was poorly reactive to C4F6 as a denatured protein (Fig 5); however, mutations of K91 to N, R, or E did not abolish binding (Fig 5). To assess the importance of K91 in C4F6 binding in fixed cells, we generated a double mutation of K91E/G93A fused to YFP and expressed it in CHO cells. At 48 hours post-transfection, 95% of cells expressing the K91E/G93A variant showed C4F6 reactivity (Fig 6). Collectively, these data indicate that the Lys at position 91 is not required for C4F6 binding. Thus, the lack of binding of the K91G variant by C4F6 likely reflects a consequence of a change in the flexibility of this segment of SOD1 that lowers C4F6 binding.

To further explore the tolerance of SOD1 for mutations at position K91, we tested two additional K91 variants (K91N and K91R) for inclusion formation and C4F6 binding. Neither of these mutants produced inclusions at a high frequency (S2 Fig; Table 1). Among the series of mutations a K91, the K91N mutation emerged as the variant that most often showed reactivity to C4F6 (S2 Fig; Table 1). Overall, our data show that mutations at surface exposed Lys residues K30, K36, K39, K75, and K91 that eliminate positive charge do not produce sufficient conformational changes to evoke inclusion formation, but a subset of these mutations evoke moderate C4F6 binding (Table 1).

## Discussion

Using a visual read-out for mutant SOD1 inclusion formation and an antibody-based approach to detect altered folding, we examined relationships between loss-of-charge at positively charged positions in hSOD1 and the propensity to misfold and form inclusions. Mutations in lysine residues of hSOD1 are rare in fALS patients, and we generally found that substitution mutations in lysine were well tolerated by the protein in terms of propensity to aggregate; however, mutations at three lysine residues (K30V, K75A, K91N) modestly increased the frequency of cell reactivity to C4F6 (see Table 1), suggesting that these mutations disturbed protein conformation to some degree. Based on the behavior of the mutant SOD1:YFP fusion proteins studied here, we conclude that a simple loss of positive charge at solvent exposed Lys residues is not necessarily sufficient to alter the inherent propensity of SOD1 to form intracellular inclusions.

In prior studies we have demonstrated that cells induced to express mutant SOD1:YFP fusion genes by transient transfection produce fluorescent inclusions that is accompanied by an increase in the level of detergent insoluble SOD1:YFP [59]. Wild-type or mutant SOD1:YFP fusions proteins that show a diffuse distribution in the cytosol are readily released into cell medium by treatment with saponin, whereas the inclusion structures (when present) remain with the cell after saponin [59]. Collectively, these prior studies indicate that a diffuse distribution of SOD1:YFP in cell cytosol is associated with high mobility and solubility. Thus, the lack of inclusion formation by these Lys mutants of SOD1 fused to YFP suggests that simply losing a positively charged amino acid at any one of the four Lys residues examined here is not sufficient to cause the same degree of misfolding as occurs by mutations associated with ALS.

We used an immunological tool to assess the impact of mutations at Lys residues on SOD1 folding that involves assessing reactivity to the monoclonal antibody C4F6. This antibody was raised against purified recombinant G93A protein [69]. We have previously demonstrated that multiple fALS mutant SOD1:YFP fusion proteins are reactive with the C4F6 antibody when used in immunostaining of fixed cells [59]. Although prior studies have described the antibody as being specific for a disease-associated, misfolded conformation [44,69], later studies, along with work described here, indicate that the epitope for the antibody is likely to be a linear segment of the protein inclusive of amino acids D90 and D96 [44,71]. When used for immunostaining of fixed cultured cell models, this linear epitope is largely inaccessible in WT SOD1; whereas the epitope is more accessible in SOD1 harboring ALS-associated mutations [44,71]. In cells expressing G93A SOD1, ~90% of the cells are C4F6 reactive. Notably, the C4F6 antibody demonstrates more intense immunoreactivity for G93A variant SOD1 over WT, or any other FALS substitution at Gly 93, in immunoblots of SDS-denaturing PAGE. This finding implies that the Ala at position 93 is an integral component of the epitope and thus, the high degree of C4F6 immunoreactivity in fixed cells expressing the G93A variant may be due to both loss of native structure and higher avidity for SOD1 when position 93 is Ala. Still, as compared to WT-SOD1:YFP, which rarely produces cells that are C4F6 reactive (Table 1), some of the mutants tested (K30V, K75A, and K91N) produced moderate to modest frequencies of C4F6 reactive cells (Table 1). These data suggest that the mutations at surface exposed Lys residues that we have analyzed may modestly alter folding without inducing inclusion formation.

### Conclusions

Although the nature of the toxic form of mutant SOD1 in fALS remains imprecisely defined, it is clear that one consequence of disease-causing mutations in hSOD1 is to destabilize normal structure in a manner that facilitates aberrant homotypic self-assembly into higher order structures [48,72]. In the present study, we asked whether mutations that reduce positive charge would potentially induce inclusion formation by reducing repulsive forces as SOD1 aberrantly assembles into aggregates. Our data indicate that mutations at Lys 30, 36, 75, and 91 that would reduce surface positive charge are relatively well tolerated. Our data may explain why mutations at Lys residues are rarely found in ALS patients.

## Supporting information

S1 Fig Image of predicted 3D structure of holo human SOD1 highlighting the positions of the R-groups for the four Lys residues mutated in this study. The R-groups for all 4 of these Lys residues are predicted to project into the solvent. For positions 30 and 36 the R-groups project out of the plane of the image towards the viewer. Images were captured in Cn3D4.3.1 (https://www.ncbi.nlm.nih.gov/Structure/CN3D/cn3dwin.shtml).
(TIF)

S2 Fig C4F6 reactivity to cells expressing K91G, K91N, K91R, or K91E mutants of SOD1. CHO cells were transfected with plasmids for each SOD1:YFP variant and after 48 hours the cells were fixed and immunostained as described in Methods. One image of direct YFP fluorescence was captured before a second image of C4F6 immunoreactivity (red) was captured, using a conventional epifluoresence microscope (20x magnification). Cells transfected with WT-SOD1:YFP serve as a negative control and cells transfected with G93A-SOD1:YFP serve as a positive control.
(TIF)
