# Supplementary figures and images for: Loss of charge mutations in solvent exposed Lys residues of superoxide dismutase 1 do not induce inclusion formation in cultured cell models
Source: PLoS One. 2018 Nov 6;13(11):e0206751. doi: 10.1371/journal.pone.0206751 (PMC6219784; doi:10.1371/journal.pone.0206751)

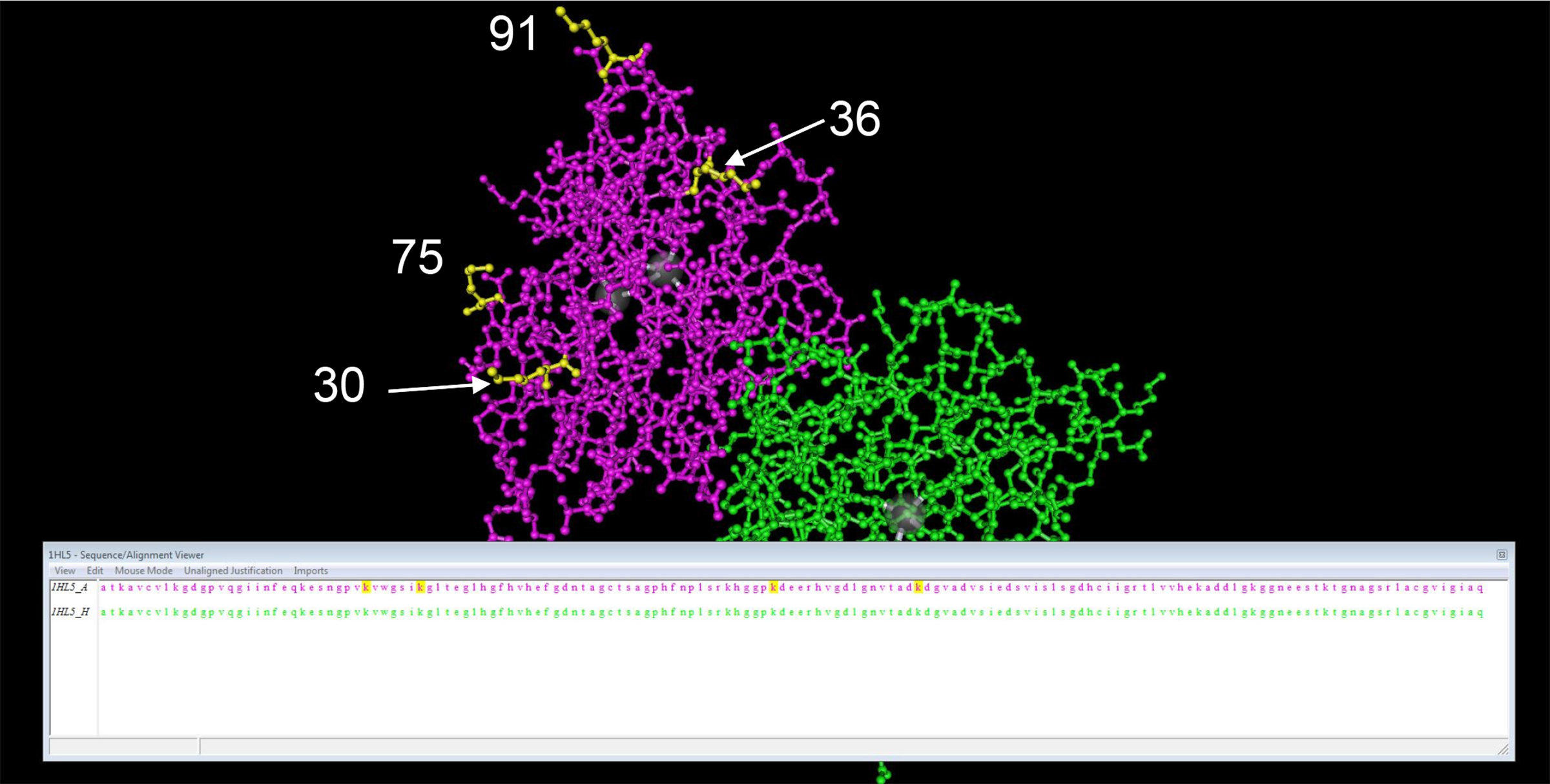

Supplement: S1 Fig — The R-groups for all 4 of these Lys residues are predicted to project into the solvent. For positions 30 and 36 the R-groups project out of the plane of the image towards the viewer. Images were captured in Cn3D4.3.1 (https://www.ncbi.nlm.nih.gov/Structure/CN3D/cn3dwin.shtml). (TIF) [file pone.0206751.s001.tif]

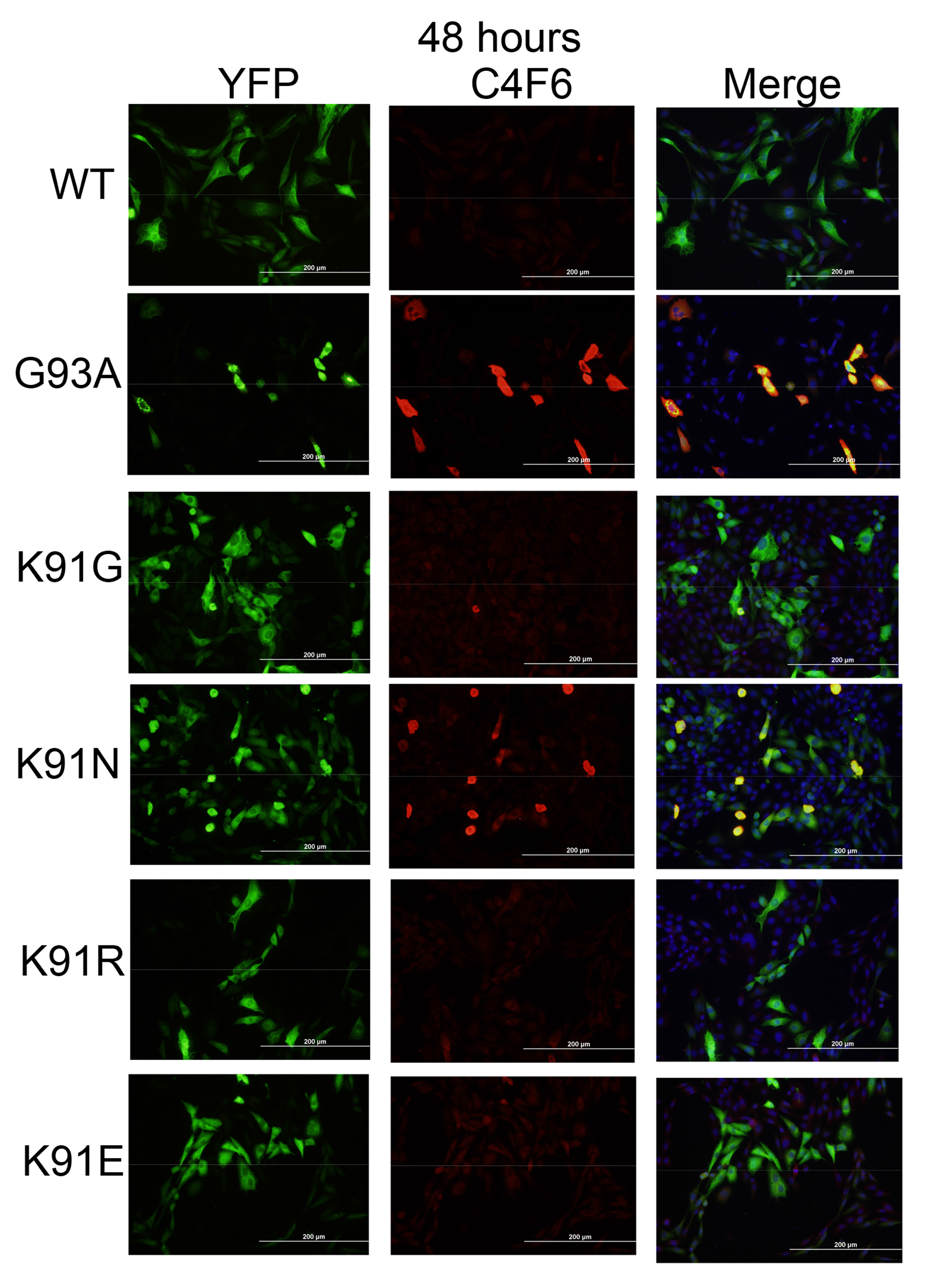

Supplement: S2 Fig — CHO cells were transfected with plasmids for each SOD1:YFP variant and after 48 hours the cells were fixed and immunostained as described in Methods. One image of direct YFP fluorescence was captured before a second image of C4F6 immunoreactivity (red) was captured, using a conventional epifluoresence microscope (20x magnification). Cells transfected with WT-SOD1:YFP serve as a negative control and cells transfected with G93A-SOD1:YFP serve as a positive control. (TIF) [file pone.0206751.s002.tif]
